# Genetic dissection in mice reveals a dynamic crosstalk between the delivery pathways of vitamin A

**DOI:** 10.1016/j.jlr.2022.100215

**Published:** 2022-04-19

**Authors:** Jean Moon, Srinivasagan Ramkumar, Johannes von Lintig

**Affiliations:** Department of Pharmacology, School of Medicine, Case Western Reserve University, Cleveland, OH, USA

**Keywords:** intestine-specific homeodomain transcription factor, stimulated by retinoic acid 6, retinol binding protein, retinoic acid, lipids, cell signaling, transport, retinoids, vitamin A, BC, β-carotene, *Bco1*, β-carotene oxygenase-1, cDNA, complementary DNA, CRBP1, cellular RBP, *DKO*, double KO, dRA, deuterated RA, eWAT, ependymal white adipose tissue, ISX, intestine specific homeodomain transcription factor, LRAT, lecithin: retinol acyl transferase, RA, retinoic acid, RAR, RA receptor, RBP, retinol binding protein, RBPR2, RBP receptor 2, RE, retinyl ester, ROL, retinol, *Scarb1*, scavenger receptor class B type 1, STRA6, stimulated by retinoic acid 6, VAS, vitamin A sufficiency

## Abstract

Vitamin A is distributed within the body to support chromophore synthesis in the eyes and retinoid signaling in most other tissues. Two pathways exist for the delivery of vitamin A: the extrinsic pathway transports dietary vitamin A in lipoproteins from intestinal enterocytes to tissues, while the intrinsic pathway distributes vitamin A from hepatic stores bound to serum retinol binding protein (RBP). Previously, the intestine-specific homeodomain transcription factor (ISX) and the RBP receptor STRA6 were identified as gatekeepers of these pathways; however, it is not clear how mutations in the corresponding genes affect retinoid homeostasis. Here, we used a genetic dissection approach in mice to examine the contributions of these proteins in select tissues. We observed that ISX deficiency increased utilization of both preformed and provitamin A. We found that increased storage of retinoids in peripheral tissues of ISX-deficient mice was dependent on STRA6 and induced by retinoid signaling. In addition, double-mutant mice exhibited a partial rescue of the *Stra6* mutant ocular phenotype. This rescue came at the expense of a massive accumulation of vitamin A in other tissues, demonstrating that vitamin A is randomly distributed when present in excessive amounts. Remarkably, provitamin A supplementation of mutant mice induced the expression of the RBP receptor 2 in the liver and was accompanied by increased hepatic retinyl ester stores. Taken together, these findings indicate dynamic crosstalk between the delivery pathways for this essential nutrient and suggest that hepatic reuptake of vitamin A takes place when excessive amounts circulate in the blood.

## Graphical abstract

**Figure:**
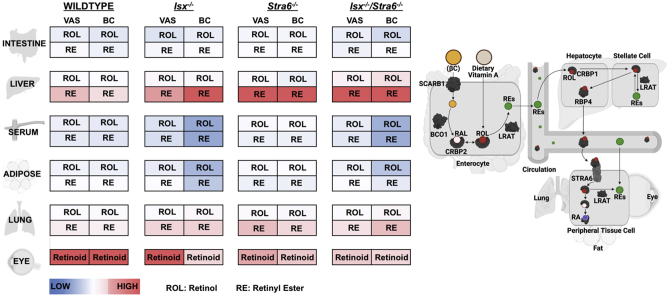

Mammals convert vitamin A (all-*trans*-retinol, ROL) into two biologically active metabolites, 11-*cis-*retinaldehyde (11cisRAL) and all-*trans*-retinoic acid (RA). 11cisRAL is the chromophore of visual pigments in photoreceptors of the retina (1). RA is a hormone-like compound and binds to RA receptors (RARs) (2). These ligand-activated nuclear receptors form heterodimers with retinoid X receptors and control expression of target genes involved across a plethora of physiological processes (3).

To support these processes, vitamin A precursors such as retinyl esters (REs) and β-carotene (BC) must be rendered available from the diet, transported, and enzymatically metabolized (1). In intestinal enterocytes, freshly absorbed vitamin A is packaged as RE into chylomicrons and secreted into the circulation. Peripheral tissues acquire retinoids from chylomicrons in a lipoprotein lipase-dependent manner (4). The large remainder in chylomicron remnants is deposited in the liver where the vitamin is stored in hepatic stellate cells (5).

Vitamin A from liver stores is secreted together with ROL binding protein (RBP) encoded by the *Rbp4* gene. In the circulation, holo-RBP forms a complex with the 55 kDa transthyretin homotetramer at a 2:1 M ratio (6). The cellular uptake of ROL from holo-RBP is mediated by a high-affinity receptor localized at the cytoplasm membrane of target tissues (7, 8, 9, 10). This receptor is encoded by the *Stra6* gene (11, 12). STRA6 facilitates the bidirectional flux of ROL between RBP and cells (13, 14). Cellular accumulation of ROL involves cellular RBP (CRBP1), encoded by the *Rbp1* gene and lecithin retinol acyl transferase (LRAT) (13, 14, 15, 16). LRAT is a microsomal membrane-anchored enzyme that converts ROL into RE by transferring palmitate from lecithin to ROL (17). A second RBP receptor encoded by the *Rbpr2* gene is expressed in the liver and intestine but is functionally less well characterized in mammals (18).

Genetic studies revealed that the extrinsic pathway for dietary vitamin A and intrinsic pathway for stored vitamin A are overlapping and partially complementary (19, 20, 21). However, it remains less well understood if crosstalk exists between them, affecting vitamin A storage and distribution. Among the key proteins in the two pathways are intestine-specific homeobox transcription factor (ISX) and STRA6. The ISX protein is the gatekeeper of the extrinsic pathway and prevents excessive provitamin A absorption and conversion of retinoids by a negative feedback loop involving retinoid signaling (22, 23). The significance of STRA6 in the intrinsic pathway for ocular vitamin A homeostasis has been highlighted under conditions of vitamin A deficiency, as circulating holo-RBP is the sole mode of transport on such restricted diets (16, 24). Previous studies also suggested that reduced clearance of circulating holo-RBP resulting from STRA6 deficiency may lead to nonspecific RA synthesis in tissues (14). Induction of *Stra6* gene expression by retinoid signaling may drive the flux of retinoids into the RE storage form to prevent excessive production of the transcriptionally active form of the vitamin (15). However, there remains a limited understanding of retinoid homeostasis, specifically the role of STRA6 during periods of vitamin A excess.

Here, we compared vitamin A metabolism in WT, *Isx*^*-/-*^ (25), *Stra6*^*-/-*^ (26), and the corresponding *Isx*^*-*/-^/*Stra6*^*-/-*^ double-mutant (double KO [*DKO*]) mice under different supply conditions for the nutrient. The aim of the study was to clarify how vitamin A distribution is affected by the different mutations and to elucidate putative interactions between the extrinsic and the intrinsic delivery pathways for vitamin A. We provide evidence for a crosstalk between the pathways and showed that STRA6 is a critical control element for vitamin A homeostasis of nonocular tissues such as the lung and spleen. We also observed that dysregulation of the extrinsic pathways caused hypervitaminosis A in peripheral tissues and was associated with circulating REs. Moreover, we demonstrated that BC supplementation to the mutant resulted in an induction of hepatic RBP receptor 2 (RBPR2) gene expression and enhanced retinoid storage. Thus, our studies provide a molecular framework for the controversial relation of the extrinsic and intrinsic delivery pathways for the fat-soluble vitamin.

## Materials and methods

### Animals, husbandry, and diets

All mouse experiments were conducted in compliance with procedures reviewed and approved by the Case Western Reserve University Institutional Animal Care and Use Committee. Studies utilized female mice that were on a C57BL/6J genetic background. *Stra6*^*-/-*^ and *Isx*^*-/-*^ mice were generated as previously described (16, 22). *Isx*^*-/-*^/*Stra6*^*-/-*^ *DKO* mice were generated in the vivaria at Case Western Reserve University. WT mice were obtained from the Jackson Laboratory. Mice were bred and raised on a standard chow diet containing ∼15,000 IU vitamin A/kg diet (Prolab RMH 3000, LabDiet, St. Louis, MO) in a room on a 12:12 h light/dark cycle with ad libitum access to food and water. After weaning, mice (n=4–5) were maintained on a diet supplemented with vitamin A (4,000 IU vitamin A, retinyl acetate) or BC (25 mg/kg) for a period of 8 weeks. Specific diets were prepared by Research Diets (New Brunswick, NJ). At the end of the dietary intervention, mice were anesthetized by using a cocktail of ketamine (20 mg/ml) and xylazine (1.7 mg/ml). Blood samples were collected via cardiac puncture. Mice were transcardially perfused with 20 ml of PBS and sacrificed by cervical dislocation. Tissues were immediately harvested for analysis or snap frozen in liquid nitrogen and stored at ˗80°C until further use.

### HPLC retinoid analysis

Retinoids were extracted from 100 μl of serum, one entire eyecup, 10 mg of liver, ∼30 mg of lung, 40 mg of gut, and white adipose tissue as previously described (27). Briefly, tissues were homogenized in 200 μl of PBS, and retinoids were extracted twice using a mixture consisting of 200 μl methanol, 400 μl acetone, and 500 μl hexane. HPLC analysis was performed on a normal-phase Zorbax Sil (5 μm, 4.6 × 150 mm) column. Chromatographic separation was achieved by isocratic flow of 10% ethyl acetate/90% hexanes. To quantify the molar amounts of retinoids, the HPLC was previously scaled with synthesized standard compounds.

### RA extraction and LC/MS analysis

RA was extracted from 100 mg of liver and lung tissue as previously described (27). In brief, samples were homogenized in 1 ml of PBS. Extraction was carried out five times using a mixture containing 3 ml of 0.025 M KOH (in ethanol), 1 ml acetonitrile, and 5 ml hexane. The top organic layer, containing no polar retinoids, was removed. Fifty microliters of 6N HCl was added to acidify the remaining ethanolic layer followed by 10 ml of hexane. The organic phase was isolated using a glass Pasteur pipette, and the solvent was evaporated under a constant stream of nitrogen. The remaining sample was reconstituted in 200 μl acetonitrile containing 0.1% formic acid. Analysis was conducted on the Hypersil GOLD 50 × 2.1 column (Thermo Fisher Scientific) by a linear gradient of water → acetonitrile (50% → 100% in 5 min followed by 100% acetonitrile for 10 min) at a flow rate of 0.3 ml/min. MS-based detection and quantification of RA was performed with a linear trap quadrupole ion trap mass spectrometer (Thermo Fisher Scientific, Foster City, CA) equipped with an electrospray ionization interface operated in the positive ionization mode. To quantify endogenous RA, a known amount of deuterated RA (dRA) standard (Cayman Chemicals, Ann Arbor, MI) was added to samples prior to extraction and LC/MS analysis. RA and dRA were detected in the selected reaction-monitoring mode using the following ion transition: 301.2 → 201.2 and 306.3 → 206.2 for RA and dRA, respectively. The relationship between the selected reaction monitoring ion intensity peaks was used to calculate the amount of endogenous RA.

### ELISA

Blood samples were assessed for RBP (R&D Systems) by ELISA according to the manufacturer’s instructions. Samples were diluted 1:100 prior to detection. 3,3′,5,5′-tetramethylbenzidine liquid substrate was used for color development and plates were read at 450 nm on a SpectraMax plate reader (Molecular Devices) immediately after terminating the reaction. RBP levels were determined based on a standard curve.

### Quantitative real-time PCR

The liver, lung, gut, and adipose tissue were flash frozen, and total RNA was extracted using the TRIzol method (Invitrogen, Carlsbad, CA). RNA was quantified using the NanoDrop ND-1000 spectrophotometer (Thermo Fisher Scientific, Waltham, MA), and complementary DNA (cDNA) was generated using the High-Capacity RNA-to-cDNA kit (Applied Biosystems, Thermo Fisher Scientific). cDNA was mixed with TaqMan Master Mix (Applied Biosystem; Thermo Fisher Scientific) and primers (Applied Biosystems) to amplify *β-actin* (Mm02619580), *Bco1* (Mm01251350), *Cyp26a1* (Mm00514486), *Isx* (Mm01243743), *Lrat* (Mm00469972), *Rbp1* (Mm00441119), *Rbp4* (Mm00803266), *Rbpr2* (Mm01345317), *Scarb1* (Mm00450234), and *Stra6* (Mm00486457). Amplification was conducted using the Applied Biosystems Real-Time PCR instrument. Gene expression levels were normalized to the expression of housekeeping gene β-actin using the ΔΔCt method. A Ct value of 35 was considered as the limit of detection.

### Immunoblotting

RBP, STRA6, LRAT, and CRBP1 proteins were assessed in tissue and serum from the various mouse lines. Tissue samples were homogenized in RIPA lysis buffer (50 mM Tris-HCl, pH 7.4, 1% NP-40, 0.5% sodium deoxycholate, 0.1% SDS, and 2 mM PMSF). The supernatant was isolated after centrifugation (20 min, 12,000 rpm at 4°C) and stored at −80°C until use. Protein concentrations for samples were determined using the BCA protein assay (Thermo Fisher Scientific). Twenty-five micrograms of protein lysates and 1 μl of serum were mixed with 2X SDS-PAGE loading buffer (1M Tris-HCl, pH 6.8, 1M DTT, and 10% SDS) and boiled for 5 min. To avoid protein aggregation in immunodetection of STRA6, samples were not boiled prior to loading unto gels. Protein samples and Precision Plus Protein Dual Color Standards (Bio-Rad) were separated by 10% (STRA6), 12% (RBP; LRAT), and 15% (CRBP1) SDS-PAGE gels and transferred to PVDF membranes (Bio-Rad). Membranes were blocked using 5% (RBP; CRBP1) and 10% (STRA6; LRAT) (w/v) nonfat dry milk dissolved in Tris-buffered saline containing 0.01% Tween-20 for 1 h at room temperature. The blots were washed and incubated overnight at 4°C with the appropriate primary antibody. As a loading control, β-actin antiserum (Cell Signaling, Boston, MA) was used at a dilution of 1:2000. For RBP immunodetection, serum albumin was used as a loading control for total protein level determinations. Antibodies to RBP (1:500, Dako, Denmark), STRA6 (1:1000, Abnova, Taipei, Taiwan), CRBP1 (1:2000, Santa Cruz Biotechnology, Inc, Santa Cruz, CA), and LRAT (1:8000, noncommercial) were used to probe the blots. Goat anti-rabbit IgG or anti-mouse IgG (Promega, Madison, WI) were employed as secondary antibodies at a dilution of 1:5000 and incubated for 1 h at room temperature. All antibodies were diluted in Tris-buffered saline containing 0.01% Tween-20. Western blots were scanned with chemiluminescence with the Odyssey Imaging System (LI-COR Biosciences).

### Statistical analysis

Statistical analyses were performed using one-way ANOVA using GraphPad Prism 8.0 software (GraphPad). An alpha level of *P* < 0.05 was considered significant. Data are expressed as mean values ± standard deviation.

## Results

### Genetic dissection of the extrinsic and intrinsic pathways for vitamin A delivery

We took advantage of ISX (25) and STRA6-deficient mouse lines (26). By conventional crossbreeding, we generated the corresponding double-mutant mice. Mice were bred on vitamin A-rich breeder chow to prevent possible developmental impairments related to the different genotypes of the mouse lines. This approach avoided confounding variables that may influence the interpretation of the dietary intervention experiment. We used female mice in the studies. At the age of 4 weeks, different mouse lines were subjected to feeding with a rodent diet (AIN-93) supplemented either with preformed vitamin A (4,000 IU/kg) (vitamin A-sufficient [VAS] diet) or with BC (25 mg/kg) (BC diet). Groups of mice (WT, *Isx*^*-/-*^, *Stra6*^*-/-*^, and *DKO*) had equal numbers and were on the same C57BL/6J genetic background. We anticipated that we will observe changes in vitamin A distribution brought upon by the mutations in the VAS diet group. The BC diet group served to elucidate the role of STRA6 under conditions of excessive supply caused by the dysregulation of the extrinsic pathway ensued by the mutation in the *Isx* gene. After 8 weeks of intervention with the two diets, mice were sacrificed in the morning since circadian rhythmicity influence several key physiological parameters. As a readout, we determined retinoid concentrations, expression of marker genes, and levels of marker proteins in different tissues. For both dietary conditions, we maintained the WT as the baseline and herein report differences as fold changes in the mutants compared to WT mice on the same diet.

### ISX controls intestinal vitamin A concentrations

**Fig. 1:**
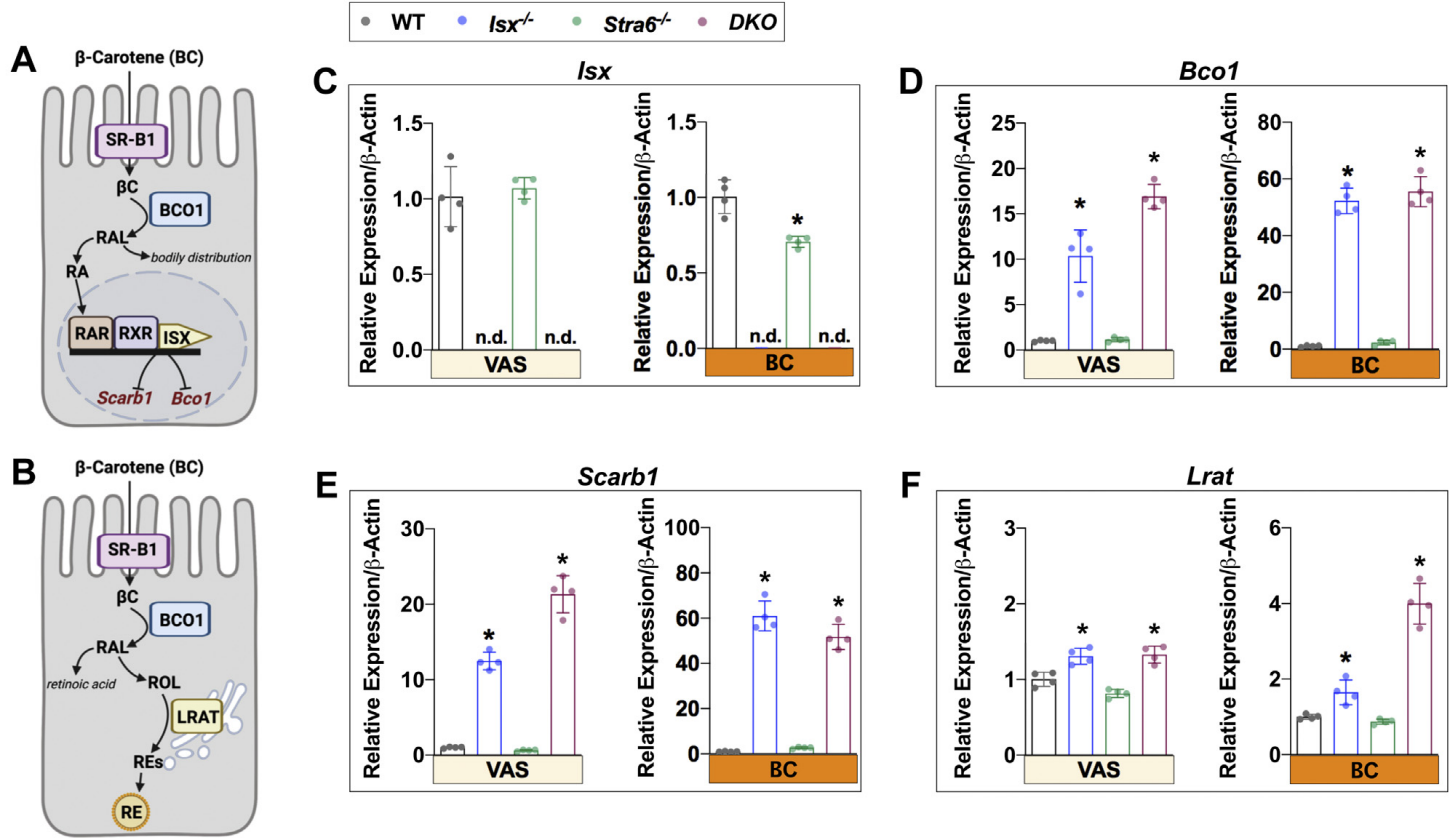
Expression of retinoid biosynthesis marker genes in the jejunum. A and B: Scheme of retinoid biosynthesis and its regulation within enterocytes. Mouse jejunum was isolated from mice (n = 4–5) maintained on either a vitamin A sufficient (VAS) or β-carotene (BC) diet for 8 weeks. Total RNA was extracted from the intestinal tissue and qRT-PCR was performed to evaluate the expression of (C) *Isx*, (D) *Bco1*, (E) *Scarb1*, and (F) *Lrat*. The data represent mean ± SD. ∗*P* < 0.05. Statistical analysis was performed using one-way ANOVA by comparing to the WT mice as the control. qRT-PCR, quantitative real-time PCR.

We first examined the effects of diet and genotype on vitamin A metabolism in the intestine (Fig. 1). *Isx* was expressed in the jejunum of WT and *Stra6*^*-/-*^ mice. As expected, its mRNA was not detected in the *Isx*^*-/-*^ and *DKO* mice. Under a VAS diet, expression levels of *Isx* mRNA were comparable in WT and *Stra6*^*-/-*^ mice, whereas *Stra6*^*-/-*^ mice displayed lower *Isx* mRNA levels on BC diet (Fig. 1C). *Bco1* and *Scarb1* mRNA levels displayed significant differences between genotypes (Fig. 1D, E). Agreeing with ISX role as a repressor, *Bco1* and *Scarb1* were significantly higher in *Isx*^*-/-*^ and *DKO* mice than in *Stra6*^*-/-*^ and WT mice. Diet also considerably impacted the expression of these genes. *Bco1* and *Scarb1* expression were elevated on VAS by ∼10- and 20-fold in *Isx*^*-/-*^ and *DKO* mice, respectively. The BC diet accentuated this pattern where *Bco1* and *Scarb1* expression were ∼40- and 50-fold higher, respectively, in *Isx*^*-/-*^ and *DKO* mice than in WT mice. Downstream of ROL formation, LRAT plays a critical role in the generation of REs, which get incorporated into chylomicrons released into circulation (27). Under both dietary conditions, the expression level of *Lrat* was elevated in *Isx*^*-/-*^ and *DKO* mice when compared to WT and *Stra6*^*-/-*^ mice (Fig. 1F).

**Fig. 2:**
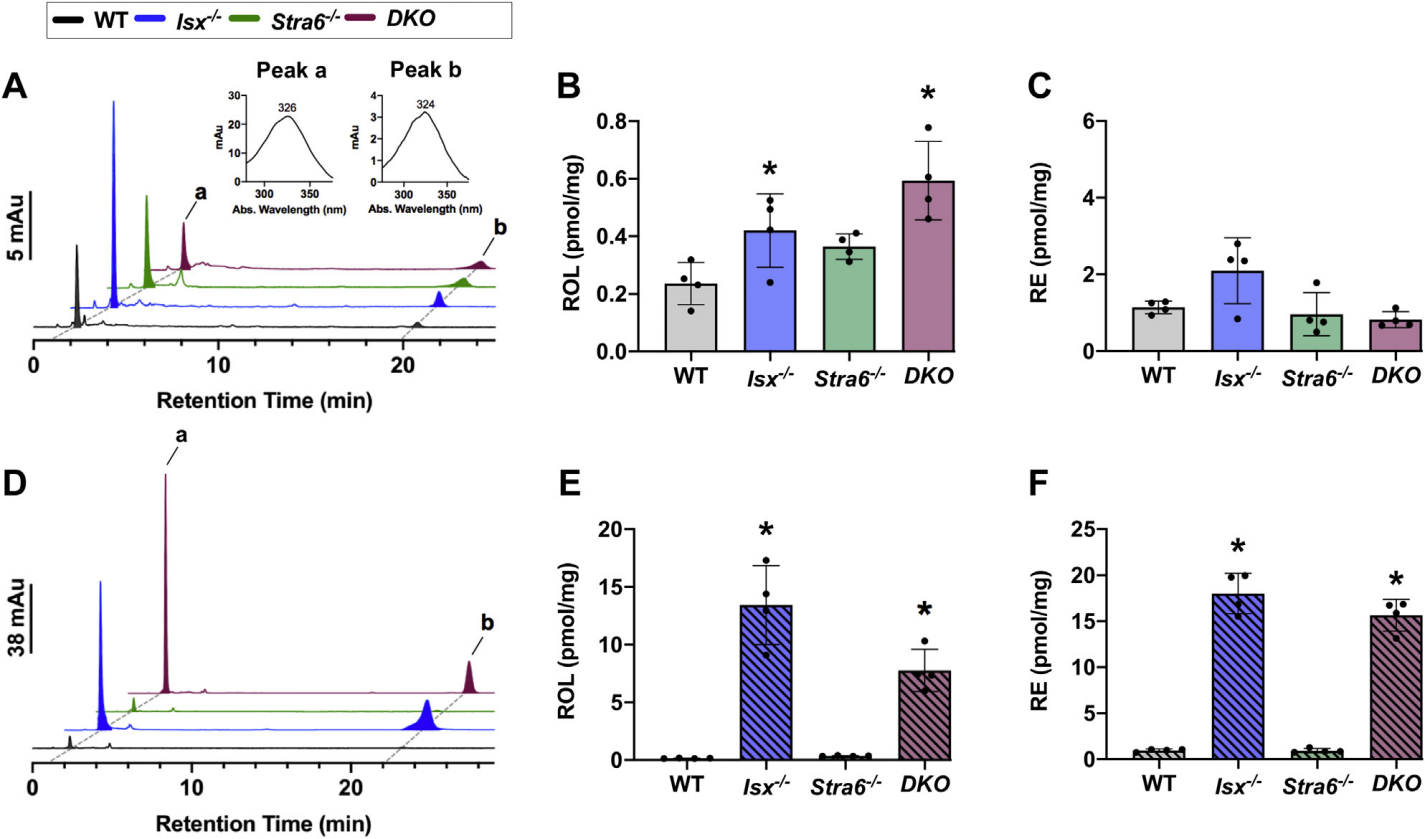
HPLC analysis for retinoids in the jejunum after VAS and BC feeding. Retinoid analysis from jejunal extracts from mice (n = 4–5) that were subjected to an 8 weeks VAS (A–C) or BC (D–F) diet. A and D: Representative HPLC traces at 325 nm. REs and ROL, *peak a* and *b*, were quantified. B and E: Retinol (ROL) levels. C and F: Retinyl esters (RE) levels. The data represent mean ± SD. ∗*P* < 0.05. Statistical analysis was performed using one-way ANOVA by comparing to the WT mice as the control.

We quantified ROL and RE concentrations in the jejunum of each mouse line by HPLC analyses (Fig. 2). With the VAS diet, the concentrations of ROL and RE were within the same range in all mouse lines (Fig. 2A–C), though *Isx*^*-/-*^ mice displayed slightly increased intestinal retinoid concentrations (Fig. 2B, C). Analysis of intestinal retinoid concentrations in the BC group revealed a shift in this pattern. ROL and RE concentrations were ∼10- and 15-fold higher, respectively, in mouse lines deficient for ISX (Fig. 2D–F). This response reflected the upregulation of *Scarb1*, *Bco1*, and *Lrat* genes that encode enzymes directly involved in BC absorption, conversion to retinoids, and esterification of retinoids. Thus, ISX deficiency led to significant enhanced utilization of BC and an excessive production of vitamin A in the intestine.

### ISX deficiency affects hepatic vitamin A metabolism and storage

**Fig. 3:**
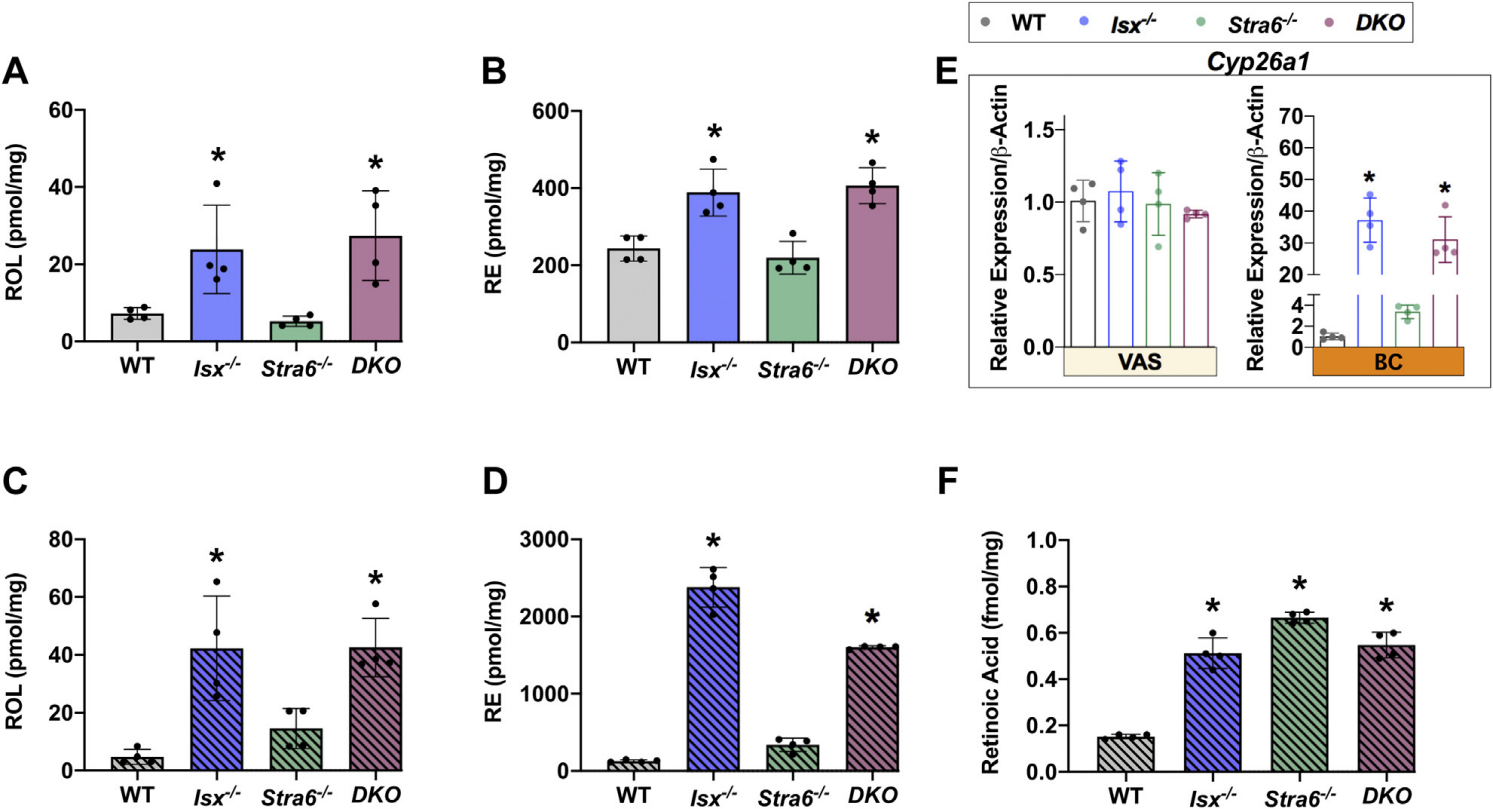
The effect of diet and genotype on hepatic retinoid concentrations. Retinoid analysis from liver extracts from mice (n = 4–5) that were subjected to an 8 weeks VAS or BC diet. A and B: Quantification of the concentration of all-*trans*-retinol (ROL) and retinyl esters (REs) under VAS conditions. C–D: Quantification of the concentration of all-*trans*-ROL and REs on BC diet. E: *Cyp26a1* mRNA levels. F: Quantification of retinoic acid levels on BC diet. The data represent mean ± SD. ∗*P* < 0.05. Statistical analysis was performed using one -way ANOVA by comparing to the WT mice as the control.

The liver is the major site for vitamin A storage and also secretes ROL complexed to RBP into the circulation. We performed HPLC analyses for hepatic retinoids in different mouse lines to evaluate how VAS and BC diets affected vitamin A storage (Fig. 3). With both diets, ROL and RE concentrations were higher in *Isx*^*-/-*^ and *DKO* mice than in WT and *Stra6*^*-/-*^ mice. The 1.5-fold increase of ROL and RE concentrations on VAS diet was surprising (Fig. 3A, B) and likely reflected the elevated *Scarb1* and *Lrat* expression in the intestine of these mice (Fig. 1). Previous studies showed that LRAT enhances absorption of preformed vitamin A (28). Moreover, increased intestinal SR-B1 is associated with overproduction of apoB48-containing lipoproteins (29) that distribute dietary lipids in the body.

On a BC diet, hepatic ROL further increased ∼2-fold in *Isx*^*-/-*^ and *DKO* mice. Notably, the concentration of REs, the storage form of vitamin A, was more than 6-fold higher in these mice (Fig. 3C, D). These findings underscore ISX as the “gatekeeper” such that the absence of ISX engenders a condition of increased absorption and conversion (Fig. 1). Remarkably, there was also a significant increase in hepatic ROL and RE concentrations in *Stra6*^*-/-*^ mice than in WT concentrations on the BC diet, though this increase was less pronounced than in ISX-deficient mice (Fig. 3C, D).

**Fig. 4:**
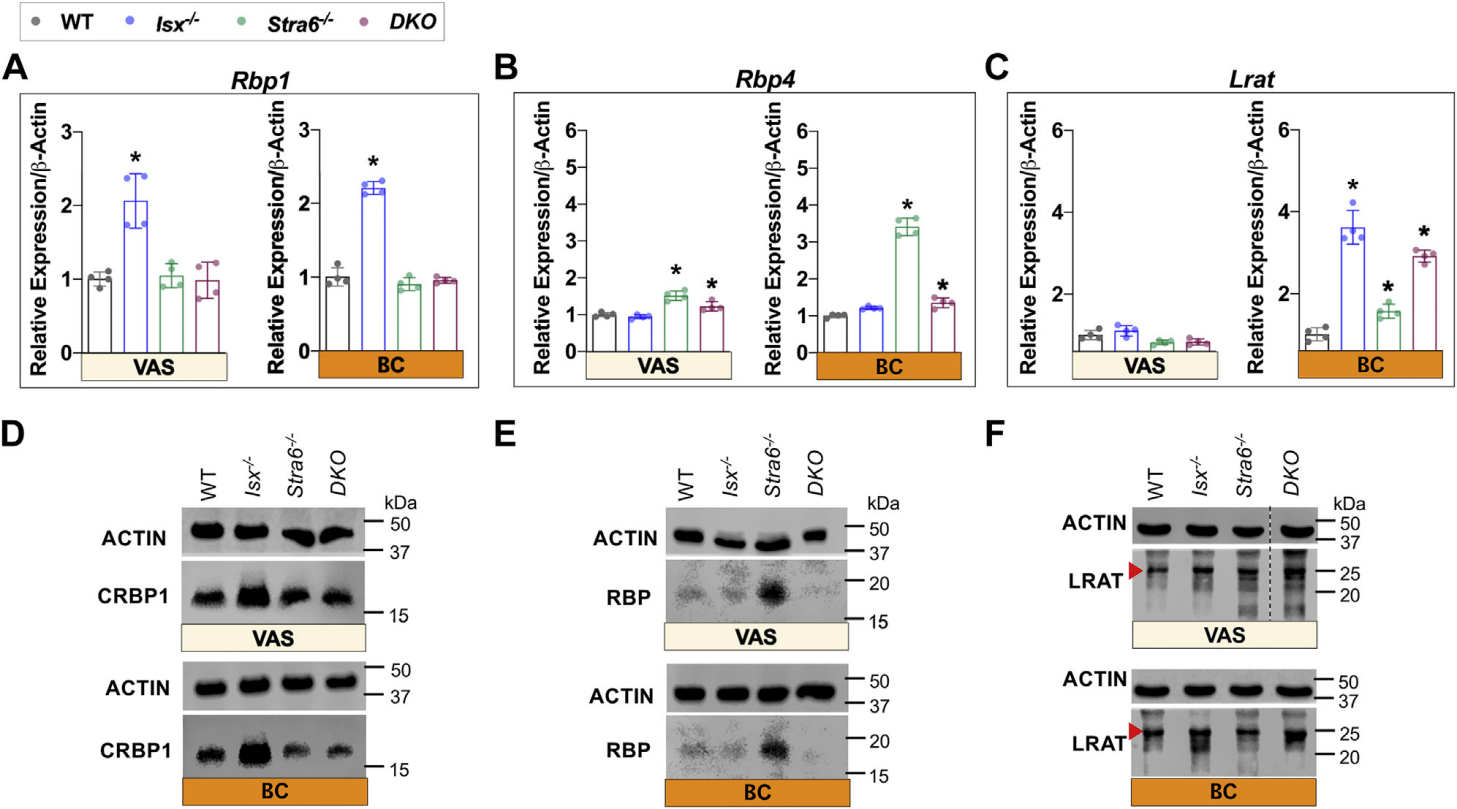
mRNA and protein expression of marker genes for retinoid metabolism in the liver. A and D: *Rbp1* mRNA and protein levels. B and E: *Rbp4* mRNA and protein levels. C and G: *Lrat* mRNA and protein levels. The data represent mean ± SD. ∗p < 0.05. Statistical analysis was performed using one way ANOVA by comparing to the WT mice as the control. Each lane (25 μg of protein for CRBP1 and LRAT and 50 μg of protein for RBP) represents an unique pool of mice (n = 4). For quantification, see supplemental Fig. S1. The *dashed line* in panel (F) indicates a splice of the blot to exclude irrelevant lanes. CRBP1, cellular retinol binding protein 1; RBP, retinol binding protein; LRAT, Lecithin Retinol Acyltransferase.

To correlate retinoid concentration with marker genes for hepatic retinoid metabolism, we determined their mRNA and protein levels (Fig. 4). Under both dietary conditions, *Isx*^*-/-*^ mice displayed significant increase in *Rbp1* mRNA and protein levels that mirrored the elevated hepatic ROL concentration (Figs. 4A,D and S1). *Rbp4* gene expression and protein levels were significantly higher in *Stra6*^*-/-*^ mice in both VAS and BC diets (Figs. 4B,E and S1). *Lrat* mRNA gene expression was comparable in different genotypes under the VAS condition but was 3- and 4-fold elevated in *Isx*^*-/-*^ and *DKO* mice on a BC diet, respectively (Figs. 4C,F and S1), and reflected the increased hepatic RE concentration.

Aside from its role in retinoid uptake, storage, and mobilization, the liver is additionally a site of RA synthesis. *Cyp26a1*, among the highly RA-inducible genes, is responsible for catabolizing RA and thereby regulating its levels (30). The mRNA levels of *Cyp26a1* were unchanged under VAS conditions but were significantly induced in *Isx*^*-/-*^ and *DKO* mice on a BC diet (Fig. 3E). We used a recently established MS method to determine if hepatic RA levels correspond to this increased gene activity in BC-supplemented mice (31). This analysis revealed that the RA concentration was more than 2-fold higher in *Isx*^*-/-*^ and *DKO* mice than in WT mice. Interestingly, *Stra6*^*-/-*^ mice also displayed a significant increase in hepatic concentration of the transcriptionally active form of the vitamin when compared to WT mice subjected to the same diet (Fig. 3F). Unchanged *Cyp26a1* mRNA expression suggested that the increase did not induce an enhanced metabolic turnover of the acidic form of the vitamin.

Taken together, the *Isx* genotype had a significant effect on hepatic retinoid metabolism by increasing the levels of ROL, RE, and RA. This was associated with increased expression levels of proteins for their biosynthesis, transport, and catabolism. The *Stra6* genotype affected *Rbp4* mRNA and protein levels. These mice displayed increased retinoid concentrations on the BC diet when compared to WT mice (Fig. 3C, D).

### Both *Stra6* and *Isx* genotype affect blood retinoid concentration

**Fig. 5:**
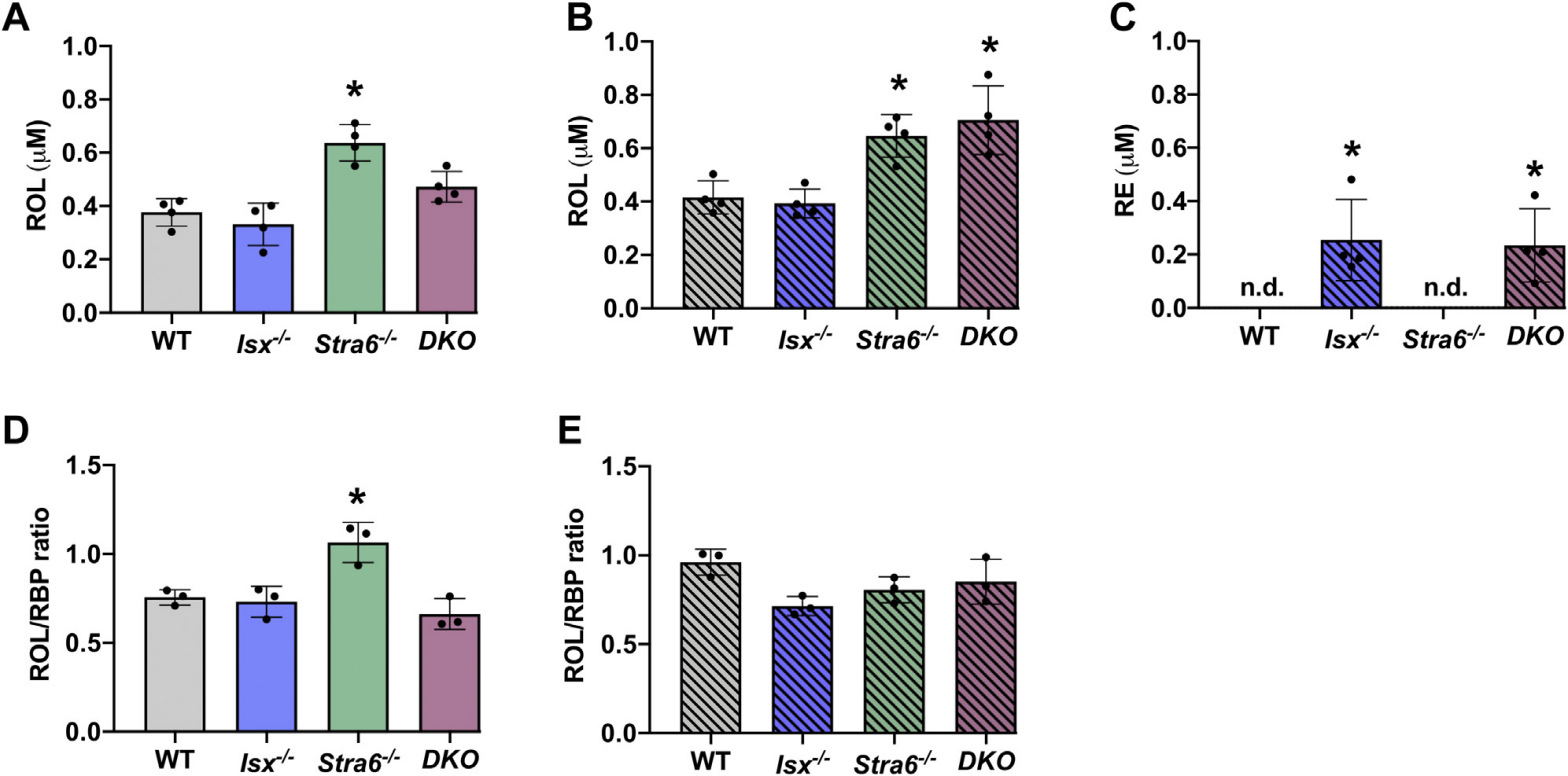
HPLC analysis for retinoids in the serum after VAS and BC feeding. Retinoid analysis of the serum from mice (n = 4–5) that were subjected to an 8 weeks VAS or BC diet. A and B: Quantification of the concentration of all-*trans*-retinol (ROL). C: Quantification of the concentration of retinyl esters (REs) on BC diet. D–E: ROL to RBP ratio on VAS (D) and BC (E) diet. The data represent mean ± SD. ∗*P* < 0.05. Statistical analysis was performed using one-way ANOVA by comparing to the WT mice as the control. RBP, retinol binding protein; RE, retinyl ester; ROL, retinol.

To study how ISX and STRA6 deficiency affected blood vitamin A and RBP concentrations, we performed HPLC and ELISA as outlined in the experimental procedures. In *Stra6*^*-/-*^ mice, serum ROL levels were significantly increased on the VAS diet when compared to WT mice. There also was an increase in ROL concentration in *DKO* mice, but it did not reach significance in statistical analysis (Fig. 5A). Differences in serum ROL concentrations between mouse lines were more pronounced on BC diet (Fig. 5B). *Stra6*^*-/-*^ and *DKO* mice displayed 20% elevated ROL levels over WT mice. The concentrations of the RBP largely correlated with the serum ROL concentration of these mice, indicating that the vitamin existed in a protein-bound form in the blood as determined by ELISA and Western blot analysis (Figs. 5D, E and S2). Thus, serum ROL and RBP concentrations increased in the absence of STRA6.

Excessive retinoid production in *Isx*^*-/-*^ and *DKO* mice on the BC diet led to an additional change in the blood chemistry. Aside from ROL, the vitamin also existed as RE in the sera (Fig. 5C). The ester form of the vitamin was not detectable in WT and *Stra6*^*-/-*^ mice as well as in the blood of all mouse lines raised on VAS diet. Together, the data indicated that the *Stra6* and *Isx* genotype both influenced blood retinoid chemistry leading to respectively higher ROL and RE concentrations in serum, especially when mice were subjected to BC feeding.

### Loss of ISX partially rescues ocular vitamin A homeostasis of *Stra6* KO mice

**Fig. 6:**
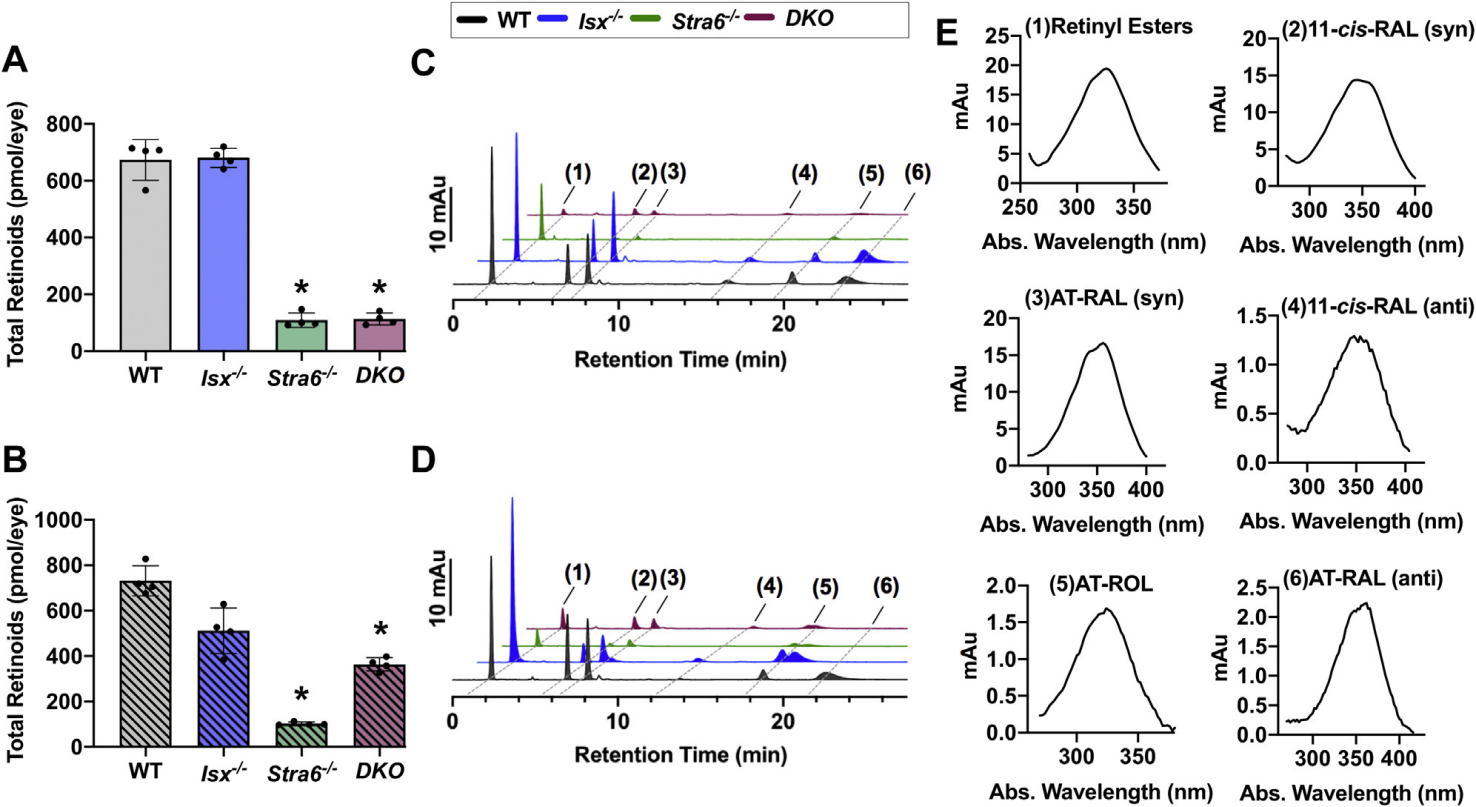
HPLC analysis ocular retinoids after VAS and BC feeding. Retinoid analysis of the eyes from mice (n = 4–5) that were subjected to an 8 weeks VAS (A) or BC (B) diet regimen. C and D: Representative HPLC traces at 325 nm. E: Spectral characteristics of the visual cycle intermediates. The data represent mean ± SD. ∗*P* < 0.05. Statistical analysis was performed using one-way ANOVA by comparing to the WT mice as the control.

We and others reported that the eyes are particularly vulnerable with STRA6 deficiency (16, 26). This is in part because STRA6 is highly enriched in the retina pigment epithelium, which supplies adjacent photoreceptors with chromophore (16, 24). For the VAS diet group, the *Stra6* genotype was the major determinate of ocular retinoid concentration. Retinoid levels were 6-fold lower in *Stra6*^*-/-*^ and *DKO* than in WT and *Isx*^*-/-*^ mice (Fig. 6A,C). In all the mouse lines, we detected different intermediates of the visual cycle, though RE levels were particularly low in *Stra6*^*-/-*^ and *DKO* mice (Fig. 6E). This observation confirmed the critical role of STRA6 to acquire the vitamin to satisfy the ocular needs for chromophore synthesis.

On a BC diet, ocular retinoid concentrations were influenced by both the *Isx* and *Stra6* genotypes (Fig. 6B,D). As before, all visual cycle intermediates were detectable in different mouse lines. We observed the highest ocular concentrations of retinoids in WT mice, whereas *Isx*^*-/-*^ mice displayed slightly lower ocular retinoid concentrations. Again, *Stra6*^*-/-*^ mice showed the lowest ocular retinoid concentrations. This indicated that regardless of the dietary source, proformed or preformed vitamin A, STRA6 deficiency renders the eyes vitamin A deficient. In contrast, *DKO* mice displayed more than 3-fold of an increase in ocular retinoids than the *Stra6* single KO mice. This finding suggested that deregulation of the extrinsic pathway for the delivery vitamin A, as seen by the high levels of RE in blood, can at least partially compensate for STRA6 deficiency.

### STRA6-dependent and STRA6-independent mechanisms contribute to vitamin A storage in the lung and spleen

**Fig. 7:**
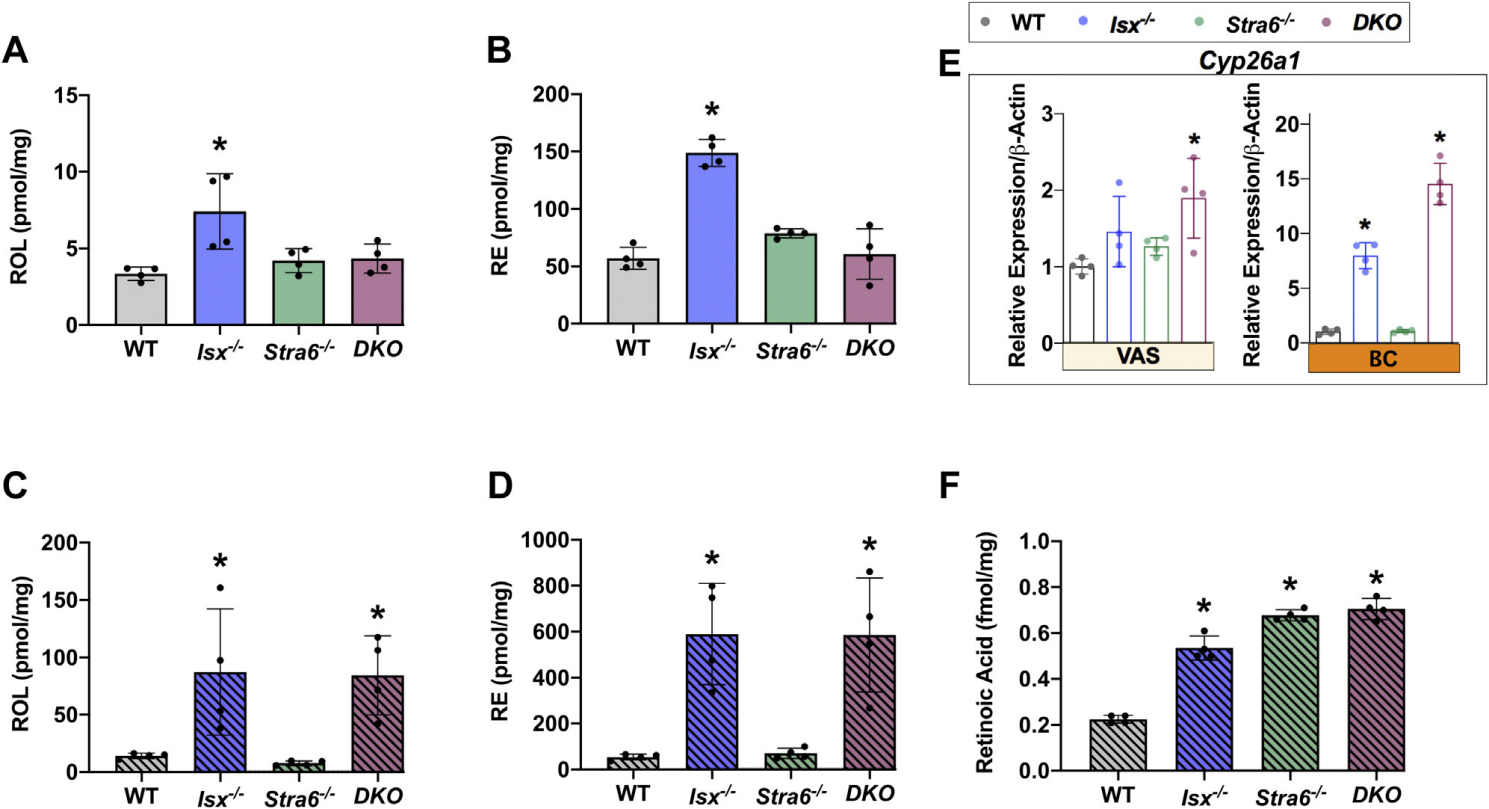
The effect of diet and genotype on lung retinoid concentrations. Retinoid analysis from lung extracts from mice (n = 4–5) that were subjected to an 8 weeks VAS or BC diet. A and B: Quantification of the concentration of all-*trans*-retinol (ROL) and retinyl esters (REs) under VAS conditions. C–D: Quantification of the concentration of all-*trans*-ROL and REs under BC conditions. E: *Cyp26a1* mRNA levels. F: Quantification of retinoic acid levels on BC diet. The data represent mean ± SD. ∗*P* < 0.05. Statistical analysis was performed using one-way ANOVA by comparing to the WT mice as the control.

The lung expresses *Lrat* and *Stra6* and has the capacity to store significant amounts of vitamin A as RE in lipid interstitial cells (15). Studies in rats observed that RA supplementation promotes storage of vitamin A in this organ during adolescence (32, 33), but it is controversial whether this enhanced storage depends on STRA6. Thus, we intended to further investigate and elucidate these inconsistencies in our study. With the VAS diet, we observed that *Isx*^*-/-*^ mice displayed significantly higher pulmonary concentrations of ROL and RE than the other three genotypes (Fig. 7A, B).

**Fig. 8:**
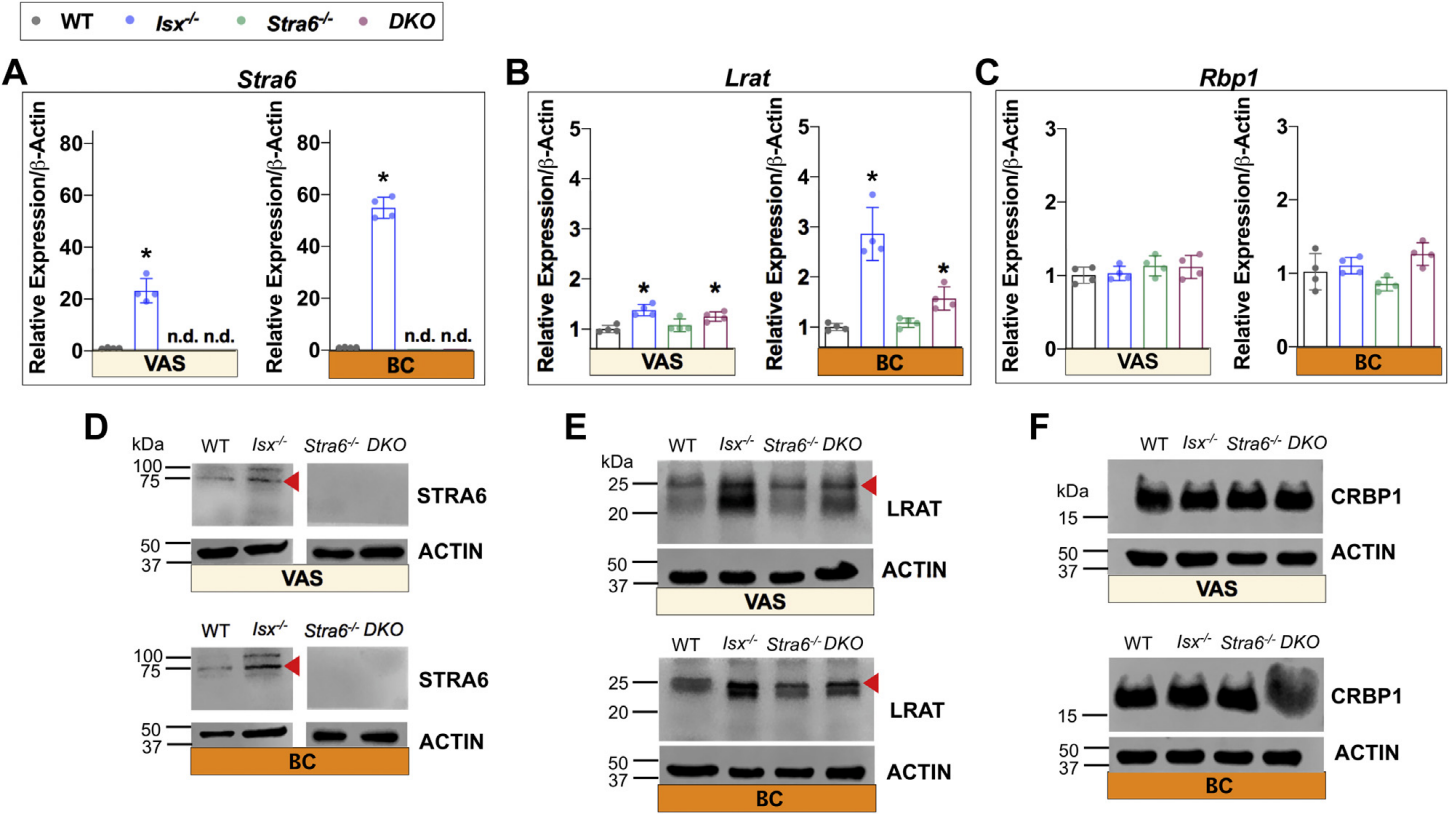
mRNA and protein expression of marker genes for retinoid metabolism in the lung. A and D: *Stra6* mRNA and protein levels. B and E: *Lrat* mRNA and protein levels. C and F: *Rbp1* mRNA and protein levels. The data represent mean ± SD. ∗*P* < 0.05. Statistical analysis was performed using one-way ANOVA by comparing to the WT mice as the control. Each lane (25 μg of protein) represents a unique pool of mice (n = 4). For quantification, see supplemental Fig. S3. CRBP1, cellular retinol binding protein 1; LRAT, Lecithin Retinol Acyltransferase; STRA6, stimulated by retinoic acid 6.

The absence of the increase in pulmonary retinoids in *DKO* mice indicated that it was dependent on a functional *Stra6* allele. Therefore, we determined the expression levels of genes involved in cellular vitamin A uptake and storage. We observed that *Stra6* gene expression was ∼25-fold higher in *Isx*^*-/-*^ mice over the level in WT mice, and this trend was mirrored in protein levels from Western blot analysis (Figs. 8A,D and S3). The *Lrat* gene acts downstream of STRA6 in cellular vitamin A uptake (14, 15), and it also showed a 1.5-fold increase when compared to WT mice. *Lrat* gene and protein expression was also elevated, albeit not significantly, in *DKO* mice, but this increase did not result in enhanced vitamin A storage likely because of the absence of STRA6 (Figs. 8B,E and S3). *Rbp1* mRNA and protein expression was comparable between all genotypes, indicating that the mRNA level of the cellular RBP did not correlate with the fluctuation in pulmonary retinoid concentrations of the different genotypes (Figs. 8C,F and S3).

With the BC diet, the effects of the *Isx* genotype on pulmonary retinoid metabolism became more pronounced than with the VAS diet (Fig. 7C, D). Concentrations of ROL and RE were increased in *Isx*^*-/-*^ mice by 6-fold and 11-fold, respectively, when compared to WT and *Stra6*^*-/*-^ mice. Our analysis revealed similar expression patterns of genes involved in vitamin A uptake homeostasis on BC diet as already observed on VAS diet. *Stra6* and *Lrat* mRNA levels increased 55-fold and 3-fold in *Isx*^*-/-*^ mice over WT levels (Figs. 8A,B and S3). As with the VAS diet, *Rbp1* mRNA and protein levels remained largely unaffected (Figs. 8C,F and S3). The elevated RE and ROL levels were supplemented with increased pulmonary RA concentrations that were determined by LC/MS analysis (Fig. 7F). This correlated well with the significant increase in *Cyp26a1* gene expression (Fig. 7E). However, the finding that *DKO* mice also displayed highly upregulated ROL and RE (Fig. 7C, D) demonstrated that the lung can store large quantities of retinoids in a STRA6-independent fashion when the extrinsic pathway for vitamin A production is dysregulated. Under this condition, vitamin A is likely acquired from circulating RE in lipoproteins (Fig. 7D) or by another STRA6-independent uptake mechanism.

We next aimed to confirm our findings from the lung in another peripheral tissue. Similar to the lung tissue, the spleen expresses both *Stra6* and *Lrat* (supplemental Fig. S5A, B). REs also can be stored in the spleen, albeit at smaller quantities. HPLC analysis demonstrated a similar trend in the retinoid profile as for the lung. *Isx*^*-/-*^ mice possessed higher concentrations of ROL and RE than the other three genotypes on a VAS diet (supplemental Fig. S4A, B), though the fold difference was not as pronounced as in the lung. When on the BC diet, the increase in levels of RE and ROL was at least 4- and 3-fold, respectively, in *Isx*^*-/-*^ and *DKO* mice (supplemental Fig. S4C, D). RA-responsive genes, *Lrat* and *Stra6*, both responded positively with the BC diet (supplemental Fig. S5A, B). Additionally, *Rbp1* mRNA expression was significantly increased in *Isx*^*-/-*^ mice under this condition when compared to the other genotypes (supplemntal Fig. S5C). Similarly, the spleen had some capacity to acquire retinoids in a STRA6-independent manner when the extrinsic pathway for vitamin A delivery was dysregulated as seen by the increased ROL and RE concentrations in *DKO* mice (supplemental Fig. S4C, D).

Taken together, dysregulation of the extrinsic delivery pathway resulted in significant upregulation of *Stra6* expression in the lung and to a lesser extent in the spleen. The increased retinoid concentration of *Isx*^*-/-*^ but not of *DKO* mice on VAS diet suggested that it was dependent on STRA6. Highly increased expression of *Stra6* and elevated retinoid concentrations were also observed in *Isx*^*-/-*^ mice supplemented with the BC diet. However, increased retinoid concentrations of *DKO* mice on the BC diet suggested that the lung also can store vitamin A independent of STRA6 when excessive amounts are provided by the diet. The spleen mirrored this phenomenon. Our data indicate both STRA6-dependent and STRA6-independent uptake. We further propose that LRAT plays a critical role for both storage mechanism as seen by its elevated expression levels in both *Isx*^*-/-*^ and *DKO* mice.

### Adipose retinoid stores are a special case

**Fig. 9:**
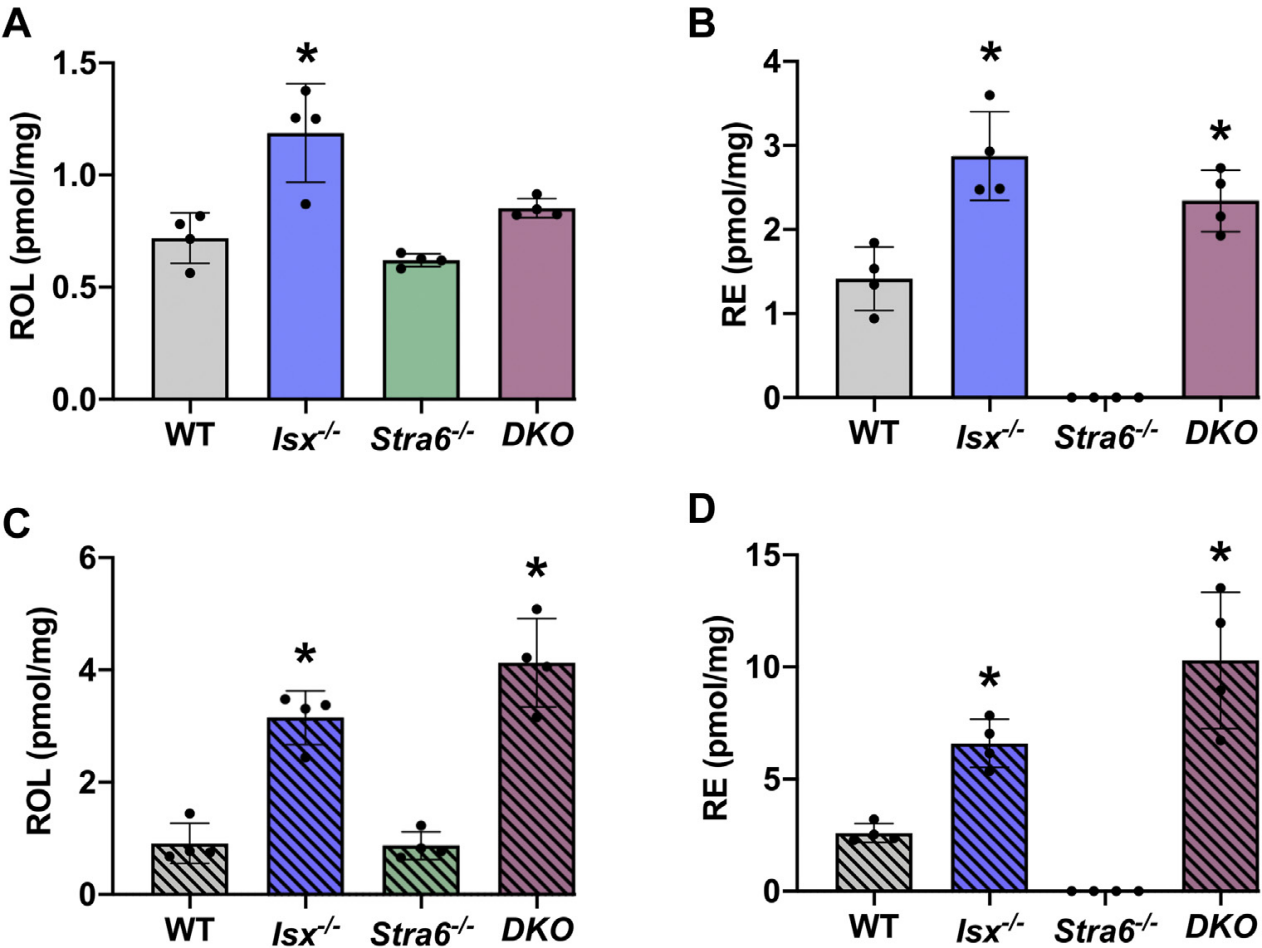
HPLC analysis of white adipose tissue after VAS and BC feeding. Retinoid analysis from splenic extracts from mice (n = 4–5) that were subjected to an 8 weeks VAS or BC diet. A and B: Quantification of the concentration of all-*trans*-retinol (ROL) and retinyl esters (REs) under VAS conditions. C–D: Quantification of the concentration of all-*trans*-ROL and REs under BC conditions. The data represent mean ± SD. ∗*P* < 0.05. Statistical analysis was performed using one-way ANOVA by comparing to the WT mice as the control.

Adipose tissue is capable of storing retinoids in the form of RE. Therefore, we determined ROL and RE in ependymal white adipose tissue (eWAT) (Fig. 9). With the VAS diet, ROL levels were elevated in *Isx*^*-/-*^ mice (Fig. 9A). RE concentrations were higher in *Isx*^*-/-*^ and *DKO* mice but below the detection levels of the HPLC system in *Stra6*^*-/-*^ mice (Fig. 9B). With the BC diet, the retinoid concentration in eWAT was influenced mainly by the *Isx* genotype of the different mouse lines (Fig. 9C, D). *Isx*^*-/-*^ and *DKO* mice, respectively, showed 4-fold and 5-fold higher ROL concentrations than WT mice and 2.5-fold and 4-fold higher RE concentrations than WT mice (Fig. 9D). Notably, ROL and RE concentrations again were markedly low in *Stra6*^*-/-*^ mice (Fig. 9C, D).

To establish whether the *Stra6* and *Lrat* genes are expressed in adipose tissues, we performed quantitative real-time PCR analysis. The C_T_ values of *Stra6* and *Lrat* were at or above the threshold of 32, indicating that the genes were not expressed in this tissue. *Rbp1* displayed a value of 25, suggesting that it is expressed at low levels in adipocytes (supplemntal Fig. S6) as previously reported by others (34). This analysis suggested that the minimal concentration of retinoids observed in *Stra6*^*-/-*^ mice on both diets is rather caused by reduced uptake from apo48-containing lipoproteins rather than by a loss of STRA6 function in adipocytes. This assumption is further corroborated by the observation that *DKO* mice displayed the highest retinoid concentration of all mice on BC diet, demonstrating that adipocytes do not depend on STRA6 for vitamin A uptake.

### Evidence for RBPR2-mediated ROL transport

**Fig. 10:**
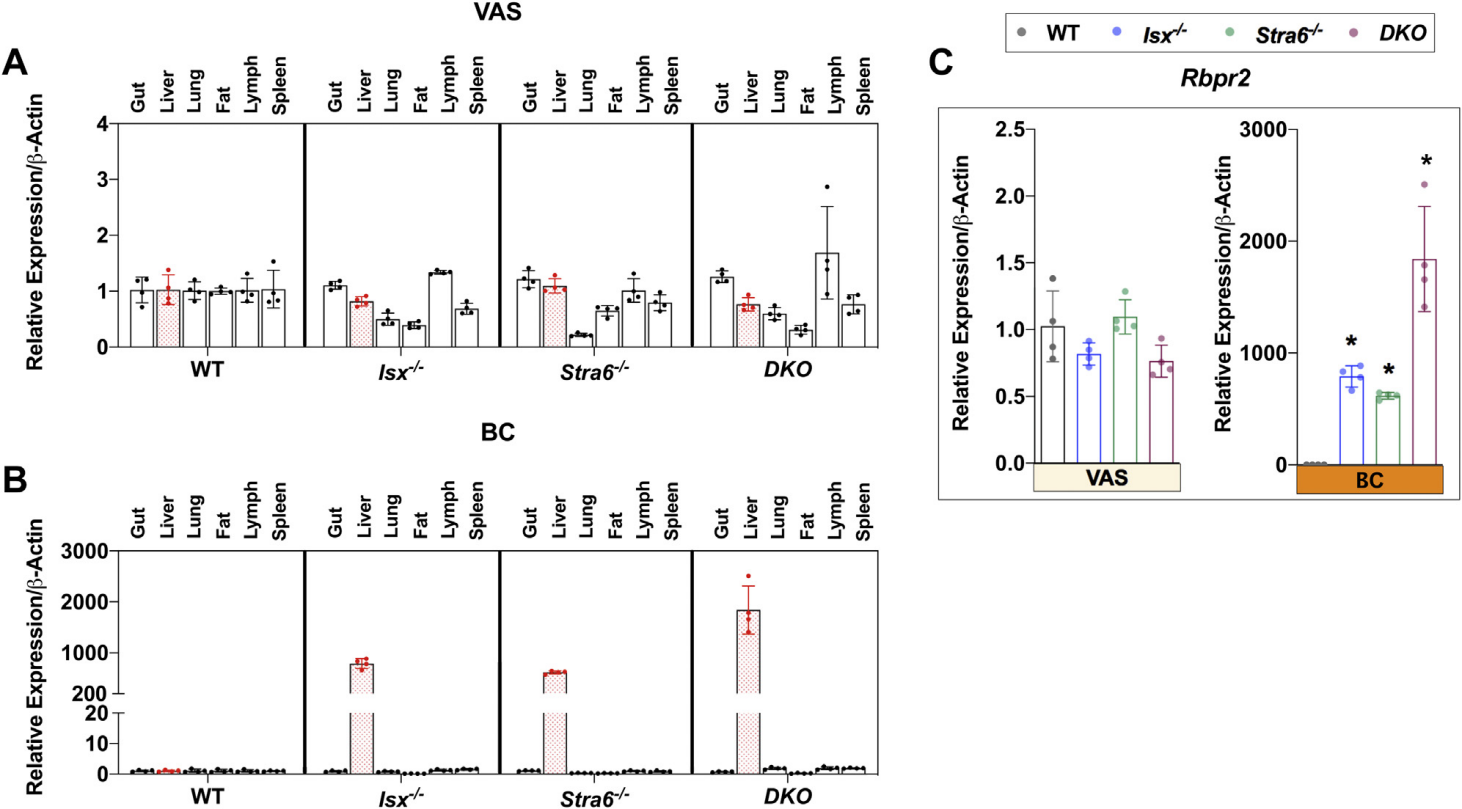
*Rbpr2* expression in tissues. RNA was extracted from tissues from mice (n = 4–5) that were subjected to an 8 weeks VAS or BC diet. mRNA levels of *Rbpr2* was determined in the VAS (A) and BC (B) dietary groups. Gene expression across tissues is presented as delta Ct. Relative changes in *Rbpr2* expression in the liver associated with diet and genotype are presented in (C).

A second RBP receptor that is mainly expressed in the liver and gut has been characterized in mice (18). However, the role of this receptor for retinoid homeostasis has not been fully elucidated. Therefore, we explored how the expression of RBPR2 is affected by different genotypes and diets. Quantitative real-time PCR analysis with mRNA preparations of different tissues revealed that *Rbpr2* is mainly expressed in the liver, intestine, and spleen of mice (Fig. 10). In other tissues, including lung and fat, *Rbpr2* mRNA expression was low, as already reported by Alapatt *et al.* (18). On VAS diet, we did not observe significant differences in the expression of *Rbpr2* mRNA between genotypes (Fig. 10A). This picture dramatically changed when we analyzed the mice on BC diet. We determined a several 100-fold increase of the expression of the second RBP receptor in the liver of all mutant mice (Fig. 10B, C).

Notably, all mutant mice displayed higher concentrations of retinoids in the liver than WT under this condition (Fig. 3C, D), though this difference in *Stra6*^*-/-*^ mice was not as pronounced as in *Isx*^*-/-*^ and *DKO* mice. Similar to STRA6, RBPR2 has been shown to bind to RBP and mediate the cellular uptake of the vitamin (18, 35). Therefore, elevated expression of *Rbpr2* and increased hepatic retinoid concentration indicate that reuptake of vitamin A occurred in the mutant mice. In STRA6 deficiency, this method of transport might be necessitated because RBP does not find an acceptor in the periphery. In ISX deficiency, the increased *Rbpr2* expression and highly increased hepatic vitamin A stores are related to the dysregulation of the extrinsic pathway that resulted in excessive vitamin A production in the gut. Under this condition, reuptake of vitamin A from circulating holo-RBP and storage in the liver may contribute to prevent accumulation of excessive amounts of the vitamin in the periphery.

## Discussion

We analyzed vitamin A homeostasis of selective tissues in WT, *Isx*^-/-^, *Stra6*^*-/-*^, and *DKO* mice fed diets formulated with preformed vitamin A or provitamin A. WT mice maintained vitamin A homeostasis in all tissues and displayed comparable concentrations of retinoids under both dietary conditions. This finding clearly indicated that WT mice can cope with different supply conditions and maintain tissue homeostasis. The analysis of the different mutant lines uncovered a dynamic crosstalk between the intrinsic and extrinsic delivery pathways for the nutrient and provided novel insights into the mechanisms that govern vitamin A homeostasis in mice.

### The diet modifies the phenotype of *Stra6*^*-/-*^ mice

*Stra6*^*-/-*^ mice displayed ocular vitamin A deficiency on diets with preformed vitamin A (24), and there was no significant improvement of this condition in mice fed copious BC. In other tissues of *Stra6*^*-/-*^ mice, we also observed significant changes in vitamin A homeostasis in response to the diets when compared to WT mice. Adipose tissues displayed highly reduced retinoid levels in STRA6 deficiency under both dietary conditions. This phenotype was previously not reported in *Stra6*^*-/-*^ mice, but these earlier studies implemented diets that provided far higher supplies of preformed vitamin A than in our study and additionally used male mice (16, 36). We rather exclude that STRA6 is directly involved in vitamin A uptake in fat. *Stra6* and *Lrat* mRNA expression in eWAT were below the threshold level of significance in all mouse genotypes. Additionally, *DKO* mice on BC diet showed the highest retinoid levels, demonstrating that adipocytes can acquire vitamin A in a STRA6-independent fashion as previously shown by others (36). Therefore, the reduced liver retinoid stores suggest that vitamin A distribution to peripheral tissues is altered in STRA6 deficiency, and adipose tissue receives significant lower supplies of retinoids. However, we did not want to exclude that STRA6 is expressed at an earlier time point in adipocytes during mouse development. A role of the RBP receptor in retinoid homeostasis and retinoid signaling during adipocyte differentiation has been previously reported (37).

Notably, *Stra6*^*-/-*^ mice displayed higher hepatic retinoid stores than WT mice on BC but not on VAS diet. The liver phenotype of *Stra6*^*-/-*^ mice was associated with increased hepatic RBP expression and elevated blood holo-RBP concentrations. Remarkably, hepatic *Rbpr2* mRNA levels increased several 100-fold on BC diet when compared to WT mice. The highly increased *Rbpr2* mRNA expression together with the increased hepatic retinoid concentration in these animals on BC diet suggested significant hepatic reuptake of circulating ROL.

### Analysis of *Isx*^*-/-*^ mice reveals a crosstalk between the delivery pathways for vitamin A

Our study revealed that the *Isx* mutation had a significant impact on vitamin A homeostasis of mice on VAS diet. *Isx*^*-/-*^ mice displayed increased hepatic and pulmonary retinoid stores under this condition. The increased stores are most likely related to elevated LRAT and SR-B1 expression in the intestine of this mouse line, affecting absorption of dietary preformed vitamin A and chylomicron metabolism (34, 38, 39). Thus, the *Isx* mutation does not only influence the metabolism of dietary pro-vitamin A but also preformed vitamin A. The increased pulmonary stores of these mice were associated with enhanced expression of STRA6 and LRAT in this tissue. A direct contribution of STRA6 to pulmonary retinoid storage in *Isx*^*-/-*^ mice was suggested by the phenotype of *DKO* mice, which lacked the increase in concentration of these stores on VAS diet. A similar trend in storage and expression of marker genes for retinoid metabolism was observed in the spleen of *Isx*^*-/-*^ mice, though the phenotype was less pronounced than in the lungs. These findings suggest that a mutation in ISX, a control element of the extrinsic pathway, affected STRA6 activity, a component of the intrinsic pathway for vitamin A delivery to the periphery. This crosstalk between the delivery pathways is explained by increased retinoid signaling in lungs and indicated that RA-mediated feedback mechanism plays a critical role in many aspects of vitamin A homeostasis. Accordingly, rats fed with RA and ROL together display increased pulmonary retinoid stores (39).

Feeding BC to ISX-deficient mice amplified their phenotype and increased vitamin A stores massively. Under this condition, LRAT expression was significantly elevated in both the liver and the lung. This observation confirmed the role of the enzyme in sequestering vitamin A in the form of RE when supplies are abundant (32, 40). Similarly, *Stra6* expression was highly elevated in *Isx*^-/-^ mice, indicating that the RBP receptor acts in conjunction with LRAT under this condition. However, uptake by STRA6 is not the only mechanism for vitamin A storage in the periphery when excessive amounts of the nutrient flood the body due to a dysregulation of the extrinsic delivery pathway. *DKO* mice displayed a high increase in retinoid stores in peripheral tissues on BC diet, though they are devoid of the RBP receptor. Notably, the dysregulation of the extrinsic pathway increased ocular vitamin A levels in *DKO* mice. However, the eyes of these mice did not reach concentrations of mice that express STRA6. In contrast, other tissues including lung, spleen, and adipose tissues of *DKO* mice displayed higher retinoid concentrations than the other mouse lines under this condition. This observation clearly demonstrated the need for a specific distribution pathway for the vitamin to satisfy ocular demands. In its absence, vitamin A is distributed nonspecifically and accumulates randomly in tissues as seen by the massive increase in lung, spleen, and fat. Remarkably, we also observed a massive increase of *Rbpr2* expression in the liver of *Isx*^*-/-*^ and *DKO* mice on BC diet. This increase in expression together with the significantly increased hepatic RE stores indicated that reuptake of circulating ROL occurred in these mice. Thus, uptake by the hepatic RBPR2 may provide a mechanism to clear excessive amounts of the vitamin from the circulation. The relevance of this hepatic reuptake of vitamin A transport deserves further investigation, including the clarification of what drives the expression of *Rbpr2* in mice on BC diet. The establishment of *Rbpr2* KO mice will be a valuable tool to further establish this pathway and to analyze its molecular details.

In conclusion, genetic dissection in mice discovered a crosstalk between the extrinsic and intrinsic pathways for vitamin A transport. Our study reveals that vitamin A homeostasis is a dynamic process that is controlled at different levels by RA-dependent feedback mechanisms. It is appropriate to assume that these mechanisms brought to light by genetic dissection of the pathway in *Isx* and *Stra6* mutant mice also play critical roles in the responses to fluctuations of the vitamin in WT mice. These fluctuations are caused by variations in dietary supply in natural environment as well as by certain disease states. A better understanding of the molecular factors that control vitamin A homeostasis, as presented here, will aid in the development of scientific-based recommendation for the intake of the essential nutrient.

## Data availability

The authors confirm that the data supporting the findings of this study are contained within the article and the supplementary information. The raw data are available upon request from the corresponding author.

## Supplemental data

This article contains supplemental data.

## Conflict of interest

The authors declare that they have no conflicts of interest with the contents of this article.

## Supplemental data

Revised Supplementary Material
