# Supplementary material for: Genetic dissection in mice reveals a dynamic crosstalk between the delivery pathways of vitamin A
Source: J Lipid Res. 2022 Apr 19;63(6):100215. doi: 10.1016/j.jlr.2022.100215 (PMC9142562; doi:10.1016/j.jlr.2022.100215)
Supplement: Supplementary file 1 — Revised Supplementary Material [file mmc1.pdf]

SUPPLEMENTARY FIGURES

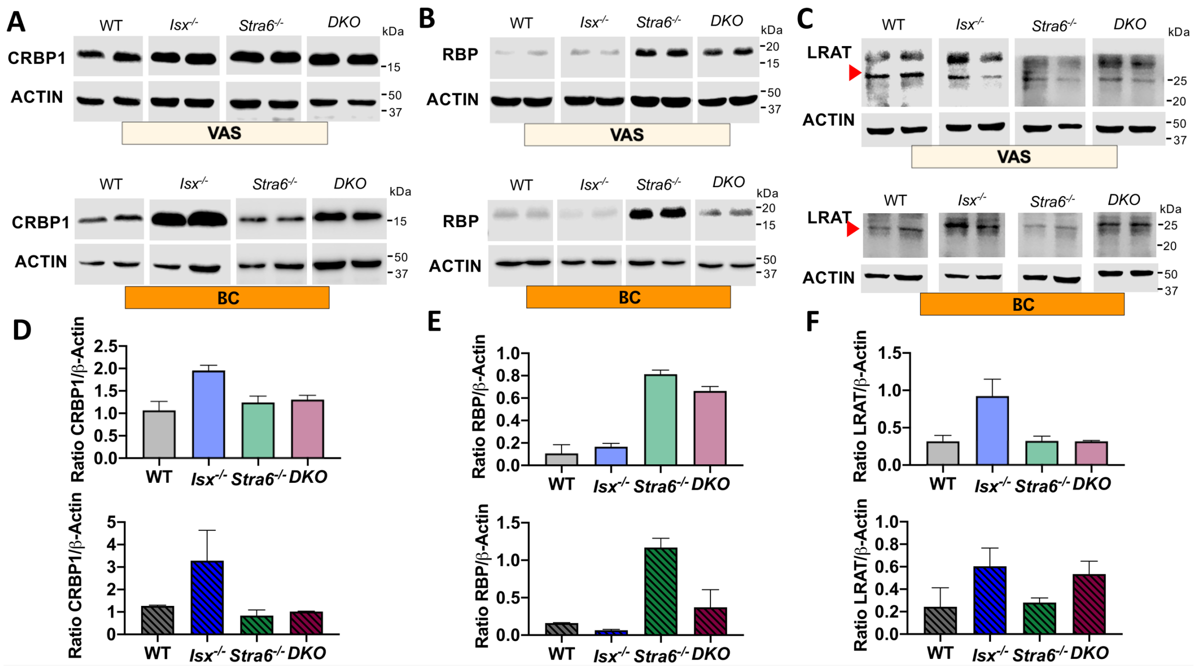

Figure S1. Western blot of proteins involved in retinoid metabolism of the liver. Western blot analysis of CRBP1 (**A**), RBP (**B**), and LRAT (**C**). Quantification of CRBP1 (**D**), RBP (**E**), and LRAT (**F**) normalized onto β-Actin. For each genotype and dietary condition, hepatic protein extracts from individual animals were mixed. Each lane (25 µg of protein for CRBP1 and LRAT and 50 µg of protein for RBP) represents a unique pool of mice (n = 4). Quantification was carried out with Image J software.

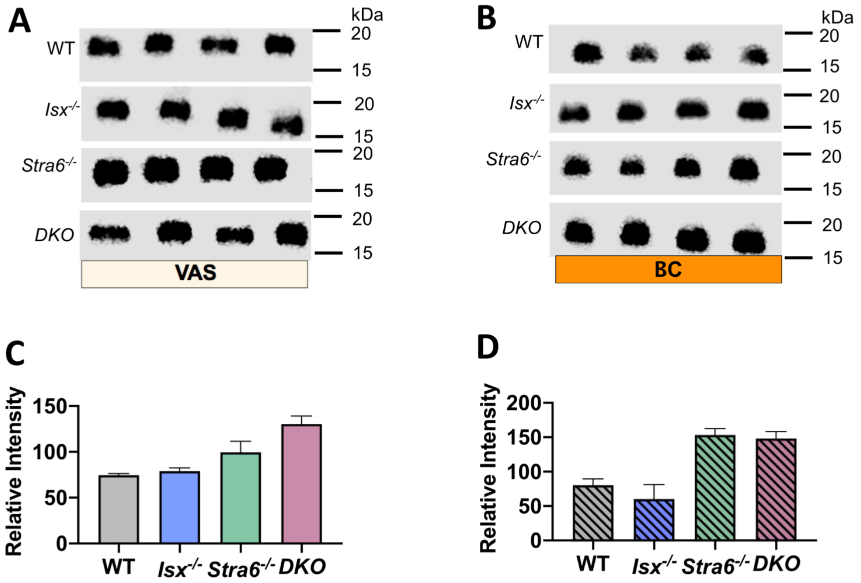

Figure S2. Western blot of serum RBP. Detection of RBP by western blot in sera of mice on a VAS (**A**) or BC (**B**) diet. Quantification of RBP under VAS (**C**) or BC (**D**) conditions. Each lane represents serum from an individual animal. Quantification was carried out with Image J software.

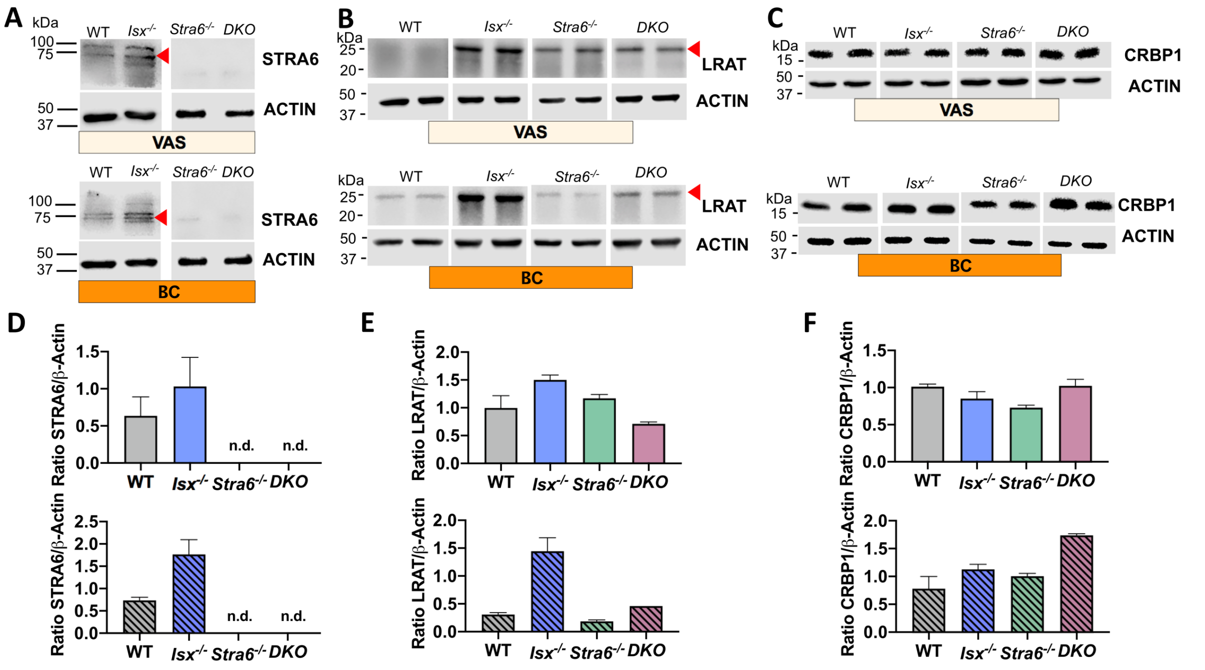

Figure S3. Western blot of proteins involved in retinoid metabolism of the lung. Detection of STRA6(**A**), LRAT(**B**), and CRBP1(**C**). Quantification of STRA6 (**D**), LRAT (**E**), and CRBP1 (**F**) normalized onto β-Actin. For each genotype and dietary condition, pulmonary protein extracts from individual animals were mixed. Each lane (25 µg of protein) represents a unique pool of mice (n = 4). Quantification was carried out with Image J software.

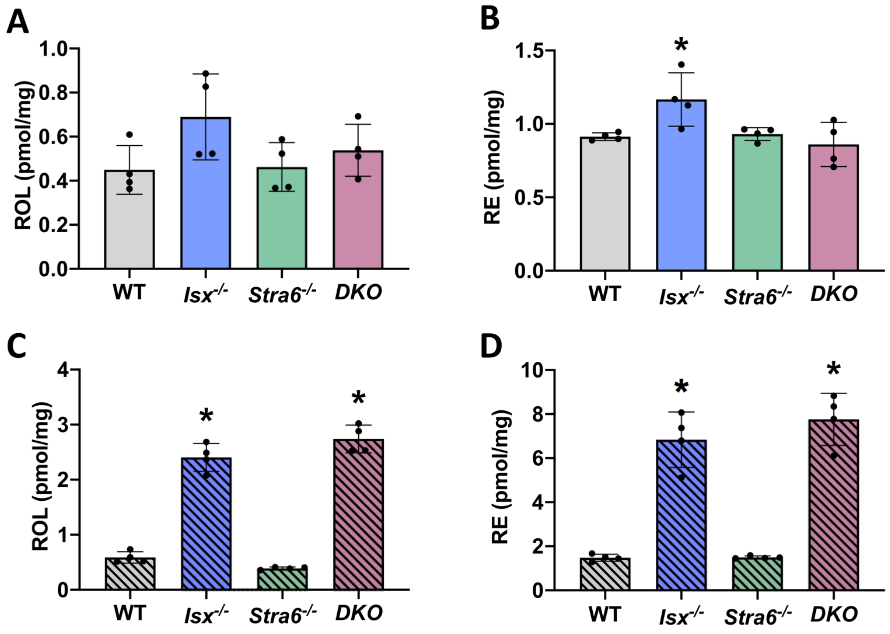

Figure S4. HPLC analysis of spleen tissue after VAS and βC feeding. Retinoid analysis from splenic extracts from mice (n = 4-5) that were subjected to 8 weeks VAS or BC diet. (**A**–**B**): Quantification of the concentration of all-*trans*-retinol (ROL) and retinyl esters (REs) under VAS conditions. (**C**–**D**): Quantification of the concentration of all-*trans*-retinol (ROL) and retinyl esters (REs) under BC diet. The data represents mean ± SD. *$p < 0.05$. Statistical analysis was performed using ANOVA by comparing to the wildtype (WT) mice as the control.

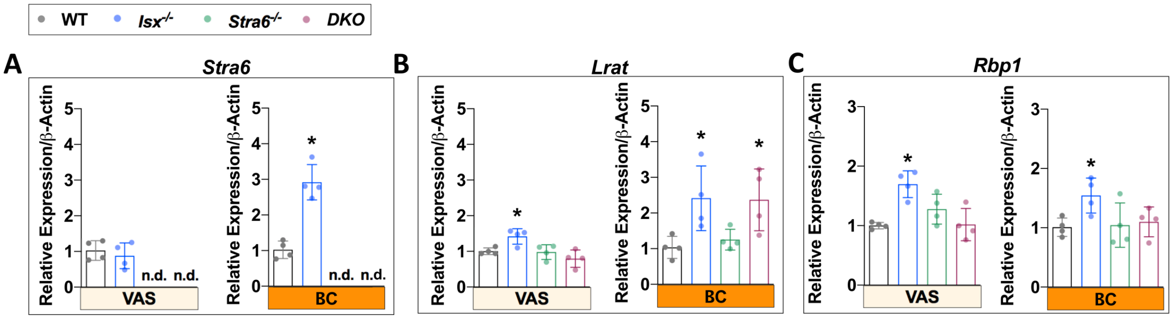

Figure S5. mRNA expression of marker genes for retinoid metabolism in the spleen. At the end of the dietary regiment, RNA from the spleen tissue was extracted to examine the expression levels of (**A**) *Stra6*, (**B**) *Lrat*, and (**C**) *Rbp1*. The data represents mean ± SD. *$p < 0.05$. Statistical analysis was performed using ANOVA by comparing to the wildtype (WT) mice as the control. *Rbp1*: cellular retinol binding protein 1; *Lrat*: Lecithin Retinol Acyltransferase; *Stra6*: stimulated by retinoic acid 6.

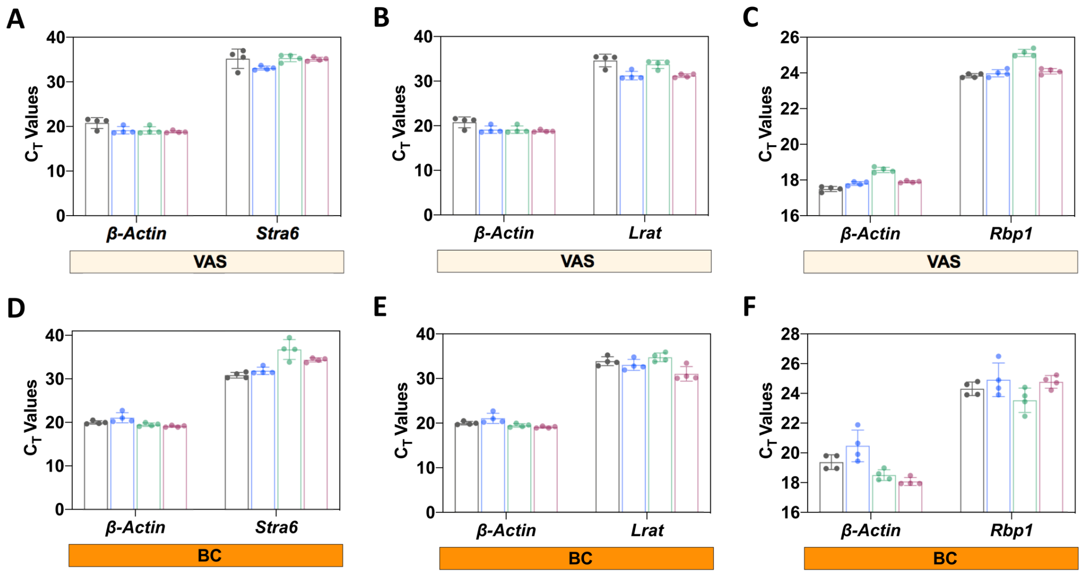

Figure S6. mRNA expression of marker genes for retinoid metabolism in white adipose tissue. RNA was extracted from adipose tissue from mice (n = 4-5) that were subjected to 8 weeks VAS or BC diet. (**A**) and (**D**), *Stra6* mRNA levels. (**B**) and (**E**), *Lrat* mRNA levels. (**C**) and (**F**), *Rbp1* mRNA levels. The data represents mean ± SD. *$p < 0.05$. Statistical analysis was performed using ANOVA by comparing to the wildtype (WT) mice as the control. *Rbp1*: cellular retinol binding protein 1; *Lrat*: Lecithin Retinol Acyltransferase; *Stra6*: stimulated by retinoic acid 6.
